# FL118, a novel camptothecin derivative, is insensitive to ABCG2 expression and shows improved efficacy in comparison with irinotecan in colon and lung cancer models with ABCG2-induced resistance

**DOI:** 10.1186/s12943-015-0362-9

**Published:** 2015-04-28

**Authors:** David Westover, Xiang Ling, Hong Lam, Jacob Welch, Chunyang Jin, Celine Gongora, Maguy Del Rio, Mansukh Wani, Fengzhi Li

**Affiliations:** Department of Pharmacology and Therapeutics, Roswell Park Cancer Institute, Elm and Carlton Streets, Buffalo, NY 14263 USA; Canget BioTekpharma, LLC, Buffalo, NY 14203 USA; Center for Drug Discovery, RTI International, Research Triangle Park, NC, 27709 USA; IRCM, Institut de Recherche en Cancérologie de Montpellier; INSERM, U896; Université Montpellier1; Institut régional du Cancer Montpellier, F-34298 Montpellier, France

**Keywords:** FL118, ABCG2, Resistance, Irinotecan, Topoisomerase I, Anticancer drug, Human tumor animal models

## Abstract

**Background:**

Irinotecan is a camptothecin analogue currently used in clinical practice to treat advanced colorectal cancer. However, acquired resistance mediated by the drug efflux pump ABCG2 is a recognized problem. We reported on a novel camptothecin analogue, FL118, which shows anticancer activity superior to irinotecan. In this study, we sought to investigate the potency of FL118 versus irinotecan or its active metabolite, SN-38, in both *in vitro* and *in vivo* models of human cancer with high ABCG2 activity. We also sought to assess the potency and ABCG2 affinity of several FL118 analogues with B-ring substitutions.

**Methods:**

Colon and lung cancer cells with and without ABCG2 overexpression were treated with FL118 in the presence and absence of Ko143, an ABCG2-selective inhibitor, or alternatively by genetically modulating ABCG2 expression. Using two distinct *in vivo* human tumor animal models, we further assessed whether FL118 could extend time to progression in comparison with irinotecan. Lastly, we investigated a series of FL118 analogues with B-ring substitutions for ABCG2 sensitivity.

**Results:**

Both pharmacological inhibition and genetic modulation of ABCG2 demonstrated that, in contrast to SN-38, FL118 was able to bypass ABCG2-mediated drug resistance. FL118 also extended time to progression in both *in vivo* models by more than 50% compared with irinotecan. Lastly, we observed that FL118 analogues with polar substitutions had higher affinity for ABCG2, suggesting that the nonpolar nature of FL118 plays a role in bypassing ABCG2-mediated resistance.

**Conclusions:**

Our results suggest that in contrast to SN-38 and topotecan, FL118 is a poor substrate for ABCG2 and can effectively overcome ABCG2-mediated drug resistance. Our findings expand the uniqueness of FL118 and support continued development of FL118 as an attractive therapeutic option for patients with drug-refractory cancers resulting from high expression of ABCG2.

**Electronic supplementary material:**

The online version of this article (doi:10.1186/s12943-015-0362-9) contains supplementary material, which is available to authorized users.

## Background

Camptothecin analogues have been used clinically to treat cancer for almost 20 years. Irinotecan (also known as CPT-11) is used in combination with other antitumor agents as a first-line therapy for metastatic colorectal cancer [1] and has a history of use as a second-line therapy in advanced gastric and non-small cell lung cancers (NSCLC) [2,3]. The second clinically used camptothecin analogue, topotecan, is approved for treatment of ovarian, cervical, and small cell lung cancers [1]. It has been established in the literature that camptothecin analogues function through inhibition of the topoisomerase I (Top1) enzyme. Camptothecin-class compounds target the DNA-Top1 covalent complex, forming a ternary complex that prevents the dissociation of Top1. This ternary complex inhibits replication and transcription and leads to the formation of double-strand DNA breaks [4,5].

Unfortunately, resistance to irinotecan and topotecan is observed in the clinic. Failure of irinotecan- and topotecan-based regimens has been hypothesized to occur through a number of different mechanisms, though only a few are supported with clinical data. *In vitro* evidence and limited clinical observations suggest mutations in the *Top1* gene decrease the affinity of the Top1 protein with clinically used camptothecin analogues [6,7]. However, based on the literature, likely a more common cause of resistance to irinotecan and topotecan is the increased expression of ATP-binding cassette (ABC), subfamily G, isoform 2 protein (ABCG2, also known as breast cancer resistance protein, BCRP), a drug efflux pump and a member of the ABC transporter superfamily [8]. A number of clinical studies revealed that failure of irinotecan and topotecan often correlates with increased ABCG2 expression [9,10]. Multiple *in vitro* studies have demonstrated that irinotecan, SN-38 (active metabolite of irinotecan), and topotecan are all substrates for ABCG2, and high expression of ABCG2 is associated with decreased intracellular accumulation of these compounds and consequentially a decrease in drug potency [11,12]. Additionally, many other anticancer agents are known ABCG2 substrates, including methotrexate [13], many anthracyclines [14], and a variety of tyrosine kinase inhibitors [15,16].

Our lab recently reported on a novel camptothecin derivative, designated FL118 [17,18]. The chemical name of FL118 is 10,11-methylenedioxy-20(S)-camptothecin, also known as 10,11-MD-CPT, MDCPT [19], and 10,11-mCPT [20] (Additional file 1: Figure S1). FL118 shows strong anticancer activity in several different cancer types *in vitro* and *in vivo* [17,18]. We have demonstrated that although FL118 is not a better Top1 inhibitor than clinically used camptothecin analogues [17,18], FL118 is able to selectively inhibit the expression of several members of the Inhibitor of Apoptosis family (survivin, XIAP, and cIAP2) and the Bcl-2 family (Mcl-1), which was demonstrated to contribute to FL118 function and anti-cancer activity [18,21]. More recent studies have further characterized the novel properties of FL118. Induction of cancer cell senescence and cell death by FL118 employs both p53-dependent and p53-independent signaling pathways, and rapid induction of wild type p53 accumulation by FL118 is largely independent of the ATM-dependent DNA damage signaling pathway but dependent on E3-competent Mdm2 [22]. Our previous studies also revealed that, while mice showed continuing body weight loss after treatment with irinotecan, body weight rapidly recovers after the completion of FL118 treatment [18,21], suggesting that FL118 possesses a more favorable toxicity profile in comparison with irinotecan.

In the present study we found that, although SN-38 and topotecan are ABCG2 substrates and fail to overcome ABCG2-mediated drug resistance, FL118 is insensitive to ABCG2 expression and effectively bypasses ABCG2 resistance. FL118 also demonstrates better antitumor efficacy than irinotecan in human xenografts with high ABCG2 expression. Additionally, we found that the relatively nonpolar nature of FL118 plays a role in bypassing ABCG2-induced resistance.

## Results

### FL118 is a more potent anticancer agent than SN-38 in NSCLC and colon cancer cell lines

**Figure 1:**
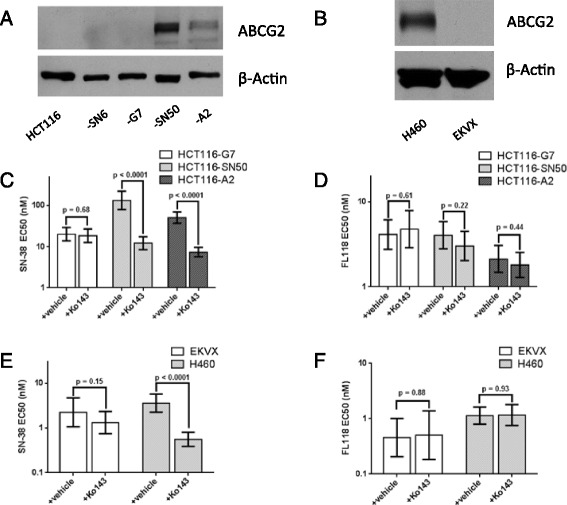
Pharmacological inhibition of ABCG2 modulates the potency of SN-38, but not FL118. **A** and **B**, Western blot analysis of ABCG2 protein expression in HCT116 colon cancer cells, drug-resistant HCT116 sub-lines **(A)**, and H460 and EKVX NSCLC cells **(B)**. **C** and **E**, dose-response curves in the presence and absence of 1 μM Ko143, an ABCG2 inhibitor, after 72 hour treatments in HCT116 sub-lines **(C)** and NSCLC cell lines **(E)**. **D** and **F**, dose-response curves in the presence and absence of 1 μM Ko143 after 72-hour treatments in HCT116 sub-lines **(D)** and NSCLC cell lines **(F)**. Viability for each dose was determined using a ViCELL XR cell viability analyzer and normalized to that of DMSO control. Error bars = SEM, n = 3 independent experiments.

The potency of FL118 versus SN-38 was compared in a panel of NSCLC and colon cancer cell lines. In each of the parental cell lines tested, FL118 was 5- to 10-fold more potent than SN-38, with EC_50_ values consistently below 1 nM (Table 1, Additional file 1: Figures S2, S3). In the four HCT116-derived camptothecin-resistant colon cancer sublines, each with mutations in *Top1* was demonstrated to decrease potency of camptothecin analogues [7], FL118 showed greater potency than SN-38 overall. Intriguingly, FL118 showed much more potency than SN-38 in sublines SN50 and A2 in comparison with sublines SN6 and G7 (Table 1). SN50 and A2 sublines highly express ABCG2, while the SN6 and G7 sublines show undetectable ABCG2 expression (Figure 1A). We assessed whether there was a difference between relative resistance (RR) for FL118 and SN-38 in HCT116 sublines. We back-transformed RR into LogRR for the purpose of statistical analysis, and found a statistically significant difference in three sublines tested (SN6, SN50, A2). Importantly, the difference between the LogRR of FL118 and SN-38 was statistically significant in both sublines with high ABCG2 expression, SN50 (p = 0.023) and A2 (p = 0.027) (Additional file 1: Table S1). Together, these data suggest that ABCG2 expression is an SN-38 resistance factor but not an FL118 resistance factor. Therefore, we hypothesized that FL118 is a comparatively poor substrate of ABCG2, and thus the potency of FL118 is not affected by ABCG2 expression.

**Table 1:** **EC50 of FL118 and SN-38 in NSCLC and colorectal cancer cell lines, including Top1 inhibitor-resistant HCT116 sub-lines**

**Cancer type**	**Cell line**	**FL118 EC50 (95%CI) (nM)**	**SN-38 EC50 (95% CI) (nM)**	**RP**	**RR(FL118)**	**RR(SN-38)**
NSCLC	H460	0.31 (0.21-0.47)	2.84 (1.98-4.09)	9.2		
	EKVX	0.56 (0.38-0.83)	2.58 (1.73-3.80)	4.6		
	A549	0.86 (0.61-1.22)	4.17 (2.30-7.57)	4.8		
Colorectal cancer	SW620	0.30 (0.19-0.46)	1.82 (1.06-3.10)	6.1		
	HCT8	0.28 (0.15-0.53)	2.21 (1.38-3.55)	7.9		
	HCT116	0.36 (0.27-0.48)	3.15 (2.42-4.10)	8.8		
	HCT116-SN6	2.28 (1.70-3.05)	7.63 (6.14-9.48)	3.3	6.3	2.4
	HCT116-G7	4.14 (2.78-6.16)	20.3 (13.9-29.6)	4.9	11.5	6.4
	HCT116-SN50	4.04 (2.79-5.86)	135 (80.4-226)	33.4	11.2	42.9
	HCT116-A2	2.13 (1.48-3.07)	51.5(37.4-70.8)	24.2	5.9	16.3

95% CI = 95% confidence interval.

RP = Relative potency, calculated by dividing the EC50 of SN-38 by the EC50 of FL118.

RR = Relative resistance, calculated by dividing the EC50 of indicated drug in resistant cell line by EC50 in parental cell line.

### Pharmacological inhibition of ABCG2 does not modulate FL118 potency

Many anticancer drugs are substrates of the ABC transporter ABCG2, which often contributes to drug treatment failure in the clinic. To determine whether FL118 potency is affected by ABCG2 activity, we utilized cell lines with varying ABCG2 protein expression to test drug sensitivity. In addition to the camptothecin-resistant HCT116 sublines discussed above, we also employed two NSCLC cell lines: H460, which express high levels of ABCG2 protein [10,23], and EKVX cells, which have undetectable levels of ABCG2 protein expression when assessed by Western blot (Figure 1B). Then, we employed a highly selective inhibitor of ABCG2, Ko143 [24], to determine whether inhibition of ABCG2 would affect FL118 potency in these cells. As expected, inhibition of ABCG2 activity by Ko143 in HCT116-A2, HCT116-SN50, and H460 cell lines, which have high ABCG2 expression, resulted in a significant increase in potency for SN-38 (Figure 1C, E) and topotecan (Additional file 1: Figure S4), indicating that they are substrates of ABCG2. In contrast, the potency of FL118 remained unchanged (Figure 1D, F) in these cell lines, suggesting that FL118 potency is unaffected by ABCG2 overexpression. Using HCT116-SN50 as a representative example, the EC_50_ of SN-38 alone was 135.1 nM, compared to 12.3 nM for SN-38 in the presence of Ko143 (p < 0.0001). In the same cell line, the EC_50_ of FL118 alone was 4.0 nM, compared to 3.0 nM for FL118 in combination with Ko143 (p = 0.22). In contrast, Ko143 did not alter the potency of either SN-38 or FL118 in HCT116, HCT116-G7, or EKVX cells that lack detectable ABCG2 (Figure 1).

### Genetic silencing or overexpression of ABCG2 does not affect the potency of FL118

**Figure 2:**
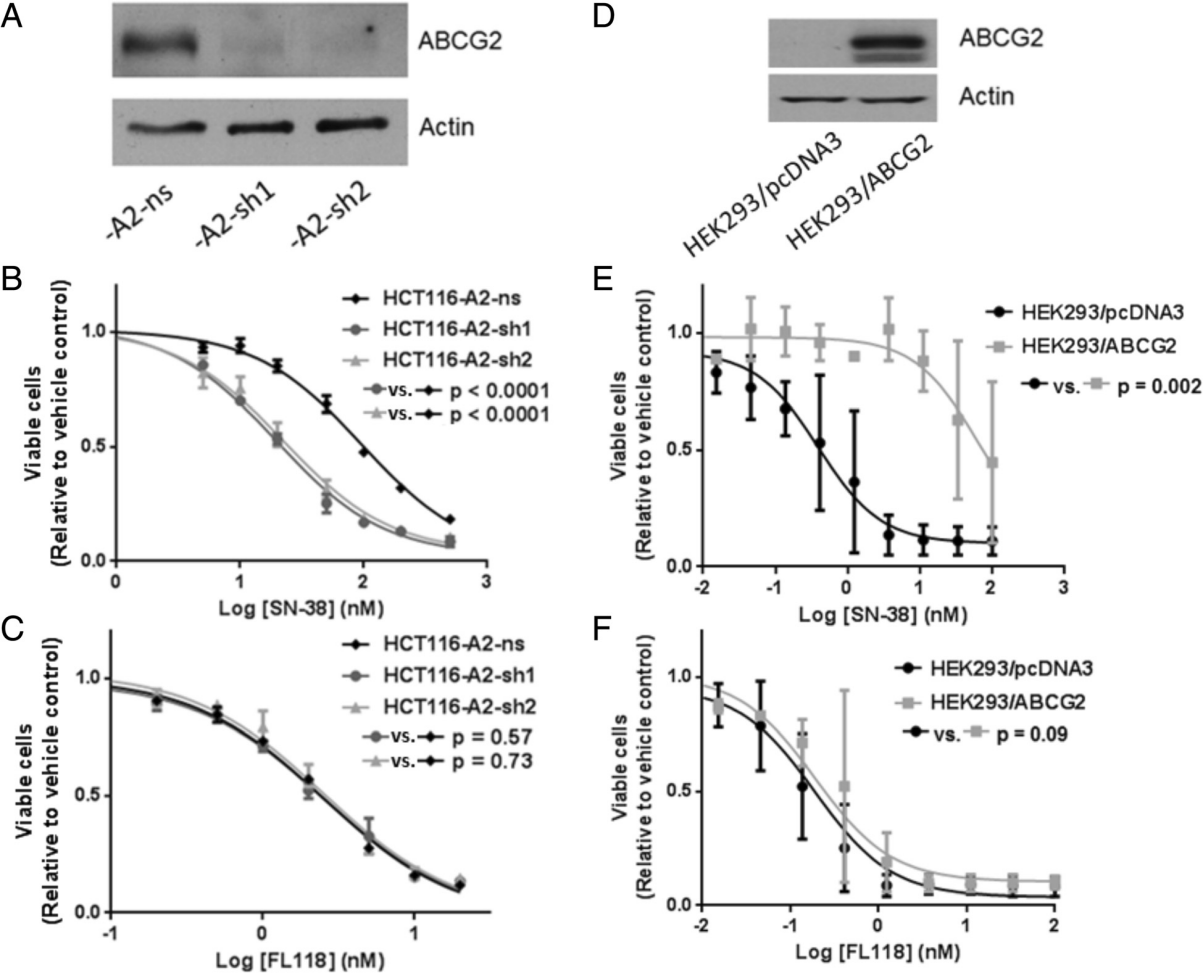
Genetic silencing or overexpression of ABCG2 demonstrates that the potency of FL118 is not affected by ABCG2. **A** and **D**, Western blot analysis of ABCG2 protein expression in HCT116-A2 cells that were stably transduced with a non-silencing shRNA control (ns) or anti-ABCG2 shRNA (sh1 = V3LHS_380805, sh2 = V3LHS_380806) **(A)**, and HEK293 cells that were stably transfected with either an ABCG2 expression vector (HEK293/ABCG2) or a corresponding empty vector (HEK293/pcDNA3) **(D)**. **B** and **C**, dose-response curves of SN-38 **(B)** and FL118 **(C)** after 72-hour treatments in HCT116-A2 cell lines. **E** and **F**, dose-response curves of SN-38 **(E)** and FL118 **(F)** after 72-hour treatments in HEK293 cell lines that were stably transfected with ABCG2 expression vector or empty vector. Viability was determined as in Figure 1. Error bars = SEM, n = 3 independent experiments.

We also employed a genetic approach through direct silencing of *ABCG2* to test the hypothesis that FL118 potency is not affected by ABCG2 expression. Two ABCG2-specific shRNAs were validated in our studies that effectively knock down ABCG2 protein expression (Figure 2A). When the expression of ABCG2 was stably knocked down in HCT116-A2 cells, the EC_50_ of SN-38 was reduced to 19.6 nM (p < 0.0001) and 22.4 nM (p < 0.0001), compared to 97.0 nM in cells transduced with a non-silencing control shRNA (Figure 2B, Additional file 1: Table S2). In contrast, consistent with pharmacological inhibition, there was no change in potency for FL118 with or without *ABCG2* silencing (Figure 2C, Additional file 1: Table S2). Next, we alternatively determined whether exogenous overexpression of ABCG2 would affect FL118 potency using a human embryonic kidney cell line, HEK293 that was stably transfected with either an ABCG2 expression vector (HEK293/ABCG2) or an empty vector (HEK293/pcDNA3) (Figure 2D). As expected, overexpression of ABCG2 decreased the potency of SN-38 from 0.39 nM in HEK293/pcDNA3 cells to 62.95 nM in HEK293/ABCG2 cells (p = 0.002) (Figure 2E, Additional file 1: Table S2). In contrast, there was no significant difference in FL118 potency in HEK293/pcDNA3 cells compared to HEK293/ABCG2 (p = 0.09) (Figure 2F, Additional file 1: Table S2). These data confirm that ABCG2 expression does not mediate resistance to FL118, suggesting that FL118 could bypass ABCG2-mediated treatment resistance.

### FL118 exhibits better antitumor activity than irinotecan and significantly extends time to progression in human xenograft models

**Figure 3:**
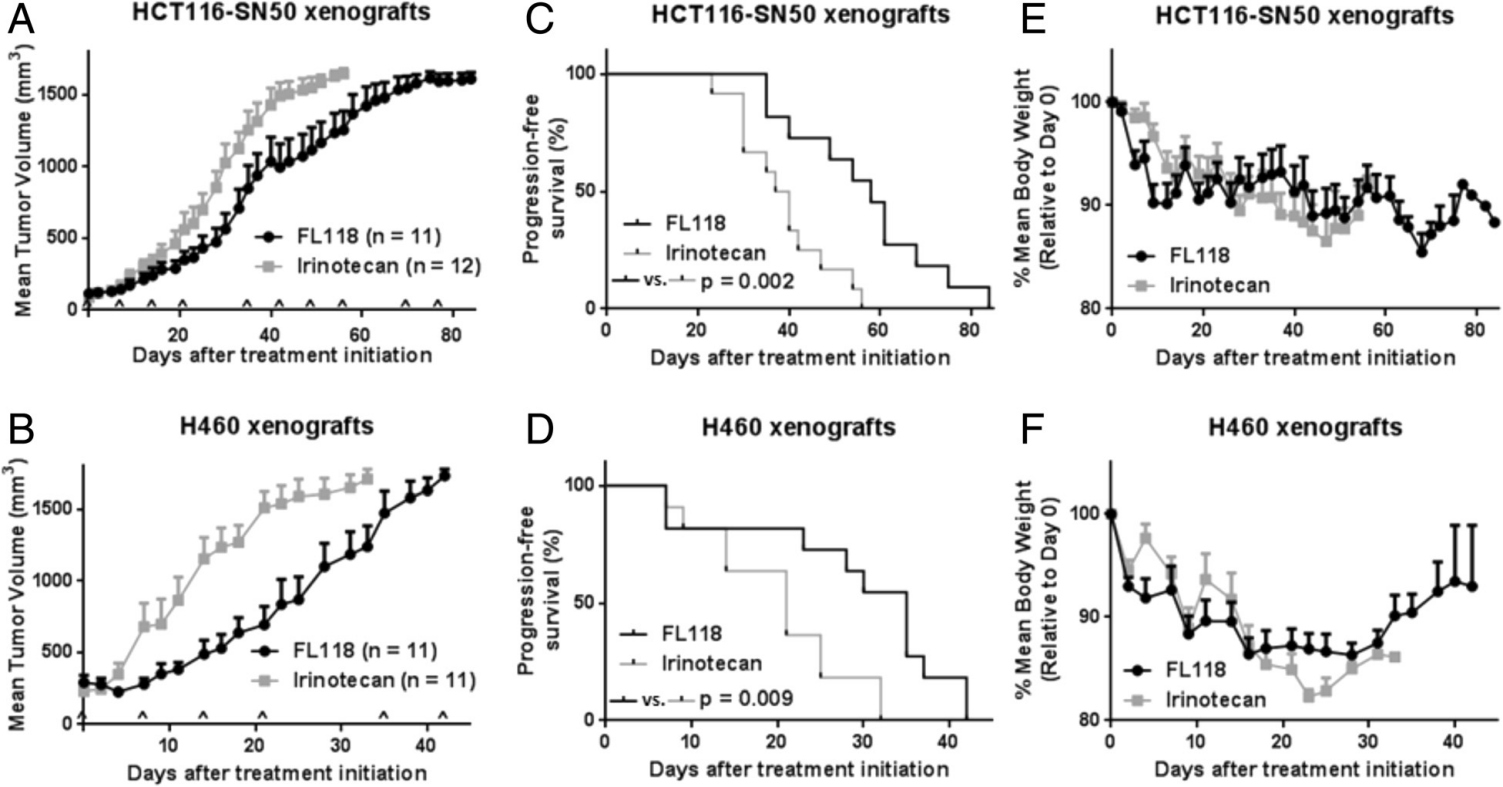
FL118 shows improved efficacy in two *in vivo* models of irinotecan-resistant cancer in comparison with irinotecan. SCID mice bearing HCT116-SN50 or H460 subcutaneous xenografts with an average volume of ~100 mm^3^ were treated via IP once per week with 100 mg/kg irinotecan or 1.5 mg/kg FL118, on a repeating treatment for 4 weeks followed by 1 week of rest. Individual animals were considered to have progressed and were removed from treatment as tumor volume reached 1500 mm^3^ or for a moribund condition. **A** and **B**, tumor growth curves for HCT116-SN50 **(A)** and H460 **(B)** xenografts during treatment with either FL118 or irinotecan. As individual mice progressed and were removed from treatment, their final tumor volume was included in the graphed average on subsequent dates. Each treatment is indicated with a caret. **C** and **D**, Kaplan-Meier chart of progression events for animals with HCT116-SN50 **(C)** and H460 **(D)** xenografts. Statistical difference in time to progression between the two treatments was determined using the log-rank test. **E** and **F**, average body weight over time during treatments. Error bars = SEM.

Next, we determined antitumor activity of FL118 versus irinotecan in HC116-SN50 and H460 xenograft models of ABCG2-mediated drug-resistant cancer. Once tumors were established and reached an average volume of ~100 mm^3^, a repeating treatment was applied intraperitoneally (IP) weekly for 4 weeks, followed by 1 week of rest. The time to progression (TTP) was used as the primary endpoint for assessment of efficacy of FL118 versus irinotecan. In both models, FL118 controlled tumor growth better than irinotecan (Figure 3A, B) and led to significantly improved TTP (Figure 3C, D). Specifically, in the HCT116-SN50 model, median TTP was 58 days for animals treated with FL118, compared to 38.5 days for animals treated with irinotecan (p = 0.002), a 50.6% increase (Figure 3C). In the H460 model, median TTP was extended from 21 days with irinotecan to 35 days with FL118 (p = 0.009), a 66.7% increase (Figure 3D). Consistent with our earlier reports, FL118 showed a tolerability profile similar to irinotecan (Figure 3E, F).

### The unique nonpolar structure of FL118 appears to play a role in FL118 bypassing ABCG2-mediated drug resistance.

**Figure 4:**
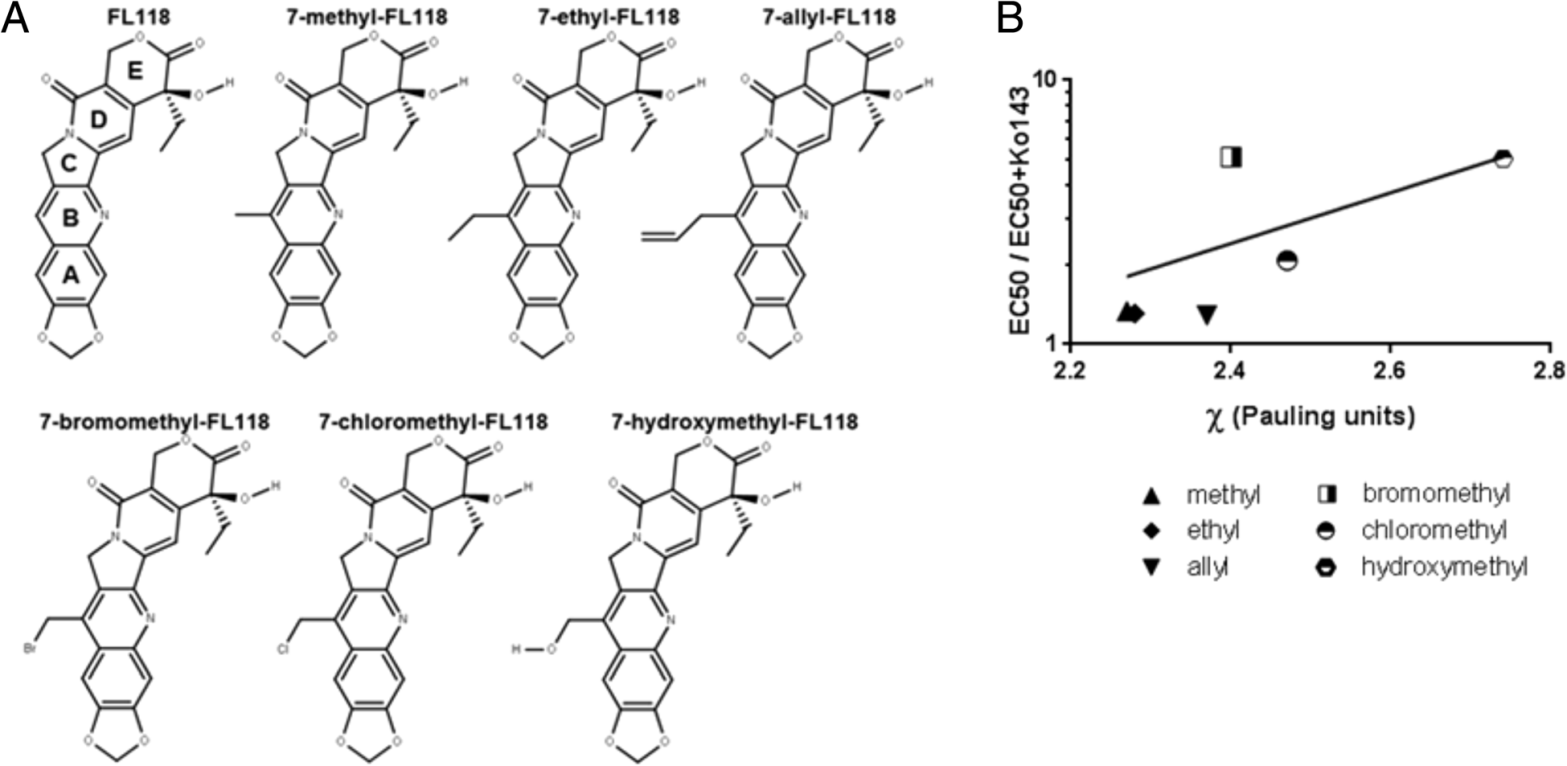
Electronegative potentials influence the affinity of the Position 7-substituted FL118 analogues for ABCG2 binding. **A**, structures of FL118 analogues with B ring substitutions. **B**, correlation between electronegativity of chemical groups (χ, Pauling units, calculated using the method of Huheey) and the ratio of EC_50_ of drug alone / EC_50_ drug + Ko143.

We next investigated the potency of several analogues of FL118 with functional groups attached to the 7-position of the B ring (Figure 4A), a position where substitutions have been shown to favorably alter drug properties [25,26], in the irinotecan-resistant sub-line HCT116-SN50. We observed little change in potency between FL118 and the FL118-derived analogues with relatively nonpolar alkyl substitutions (methyl, ethyl, and allyl) (Table 2). In contrast, addition of more polar groups (bromomethyl, chloromethyl, and hydroxymethyl) resulted in a marked decrease in potency (Table 2). Thus, adding groups to the B ring that increase the polarity of the molecule would result in decreased potency. Previous reports indicated that the addition of polar residues to the A ring of camptothecin analogues increased affinity to ABCG2 [27,28], but the effect of B ring substitutions with these types of residues remains unknown. To assess whether the polarity-related decrease in potency was at least partially dependent on ABCG2, growth inhibition was subsequently assessed in the presence and absence of ABCG2 inhibitor Ko143. Although we observed no significant change in EC_50_ for 7-methyl-FL118, 7-ethyl-FL118, or 7-allyl-FL118 with the addition of Ko143, we were able to observe a significant decrease in EC_50_ for 7-bromomethyl-FL118 (87.0 nM to 17.0 nM, p < 0.0001), 7-chloromethyl-FL118, (26.8 nM to 12.9 nM, p = 0.016), and 7-hydroxymethyl-FL118 (24.2 nM to 4.8 nM, p < 0.0001) (Table 2). Thus, the potency of the latter three FL118 analogues is affected by ABCG2 activity. We next compared the electronegativity (χ, in Pauling units) of each added functional group to the ratio of EC_50_ / EC_50_ + Ko143 (Figure 4B). Consistent with the data shown in Table 2, it was revealed that chemical groups with stronger electronegativity (bromomethyl, chloromethyl, and hydroxymethyl) show higher affinity to ABCG2 than the chemical groups with weaker or no electronegativity (methyl, ethyl, and allyl) (Figure 4B). Together, our work revealed that lack of polar functional groups on the B ring of FL118 plays a role in FL118 bypassing ABCG2-mediated drug resistance.

**Table 2:** **Electronegativities (χ, Pauling units) for added 7-position functional groups, and EC50 +/- K0143 in HCT116-SN50 cells**

**Compound**	**χa**	**χb**	**χc**	**EC50 (95% CI)**	**EC50 + Ko143 (95% CI)**	***P***	**Ratio EC50/EC50 + Ko143**
FL118	-	-	-	4.04 (2.79-5.86)	3.02 (2.08-4.49)	0.22	1.34
7-Methyl-FL118	2.27	2.47	2.40	1.49 (1.05-2.12)	1.11 (0.80-1.54)	0.20	1.34
7-Ethyl-FL118	2.28	2.48	2.43	2.43 (1.63-3.63)	1.85 (1.38-2.48)	0.22	1.31
7-Allyl-FL118	2.37	2.48	2.46	5.25 (2.71-10.2)	4.07 (2.93-5.65)	0.42	1.29
7-Bromomethyl-FL118	2.40	2.50	2.51	87.0 (53.0-142)	17.0 (13.2-23.8)	< 0.0001	5.12
7-Chloromethyl-FL118	2.47	2.53	2.54	26.8 (14.0-51.2)	12.9 (9.92-16.7)	0.016	2.08
7-Hydroxymethyl-FL118	2.74	2.59	2.52	24.2 (16.8-34.7)	4.79 (3.87-5.92)	< 0.0001	5.05

a. Huheey 1965 [37], 1966 [38], b. Inamoto 1982 [39], c. Wu 1999 [40].

95% CI = 95% confidence interval.

## Discussion

The present study expands the uniqueness of FL118 in mechanism of action to overcome drug resistance, and demonstrated that, in a panel of colon cancer and NSCLC cell lines, FL118 is more potent than SN-38. We found that, in contrast to the two clinically used camptothecin analogues (irinotecan, topotecan), which are ABCG2 substrates and unable to overcome ABCG2 resistance, FL118 potency is not affected by ABCG2 expression and can bypass ABCG2-mediated treatment resistance. This phenomenon was investigated and confirmed by multiple independent approaches to either inhibit (i.e., pharmacological and genetic inhibition) or enhance (i.e., overexpression) ABCG2 activity. These approaches demonstrated that high ABCG2 activity results in resistance to SN-38 and topotecan, but not FL118.

We also assessed the efficacy of FL118 in two distinct *in vivo* xenograft models of ABCG2-mediated drug-resistant cancer. In both models, FL118 exhibited a better ability to decrease tumor growth in comparison with irinotecan, while maintaining a tolerable toxicity profile similar to that of irinotecan. Most importantly, mice treated with FL118 showed a significant increase in time to progression (TTP) compared to mice treated with irinotecan.

Our group recently reported on the *in vivo* efficacy of FL118 in other models of human cancer, using an intravenous (IV)-compatible, Tween/polysorbate 80-free formulation [29]. In that study, we compared three schedules (every day for five injections, every other day for five injections and once weekly for 4 injections) of FL118 administration via IV administration. We found that the optimum administration of FL118 appears to be every other day. However, for this study, we opted for a weekly schedule that mimics irinotecan administration in the clinic. Therefore, while our results in this study revealed that the weekly administration of FL118 was able to significantly extend TTP in comparison with irinotecan, we predict that treating with FL118 every other day for five treatments will show even better efficacy. Additionally, in the current studies, we used IP routes instead of intravenous administration of FL118 for technical convenience. However, the Tween/polysorbate 80-free formulation of FL118 can be administrated via IV routes with increased maximum tolerated dose (MTD). This may also result in better *in vivo* tumor inhibition outcomes for FL118. We intend to perform these experiments with optimal routes and schedules in our follow-up studies as part of a broader goal of optimizing FL118 administration to prepare for clinical applications.

It was reported that the affinity of camptothecin analogues to ABCG2 is influenced by polar additions to the A ring [27,28]. We hypothesize that the polarity of B ring-substituted functional groups of FL118 may also affect ABCG2 binding. To test this hypothesis, we modeled the distinct electrostatic potential of six FL118 analogues with different chemical groups on the 7-position of the B ring versus FL118 itself, and assessed their potency using cell proliferation assays. As before, we used potency in the presence and absence of ABCG2 inhibition as a surrogate for assessing the affinity of individual drugs for ABCG2. We saw a significant change in EC_50_ in the presence of Ko143 for FL118 analogues with stronger electronegative groups, indicating that these FL118 analogues are substrates of ABCG2. We also noted that some structures with less polar functional groups (e.g., 7-ethyl-FL118) still have regions of localized electronegativity in those groups (indicated by the red gradient) (Additional file 1: Figure S5), while some structures with more polar groups lack such regions of moderate localized electronegativity (e.g., 7-chloro-FL118, Additional file 1: Figure S5). Comparing these electrostatic potential maps to our results in Table 2, we propose that these localized electronegative regions are less predictive of a compound’s affinity for ABCG2 than the Pauling electronegativity of the molecule as a whole.

We recently reported a novel IV-compatible Tween/polysorbate 80-free formulation for FL118, which could increase FL118 MTD 3-7 fold in comparison with FL118 formulated using the previous Tween/polysorbate 80-containing recipe [29]. We found that in this new formulation, while FL118 showed solubility similar to 7-bromomethyl-FL118, 7-chloromethyl-FL118 and 7-hydroxymethyl-FL118, the solubility of 7-methyl-FL118, 7-ethyl-FL118 and 7-allyl-FL118 was much poorer than FL118. Similarly, FL118 analogues with other 7-substituted nonpolar groups (e.g., cycloalkyl, aryl) also showed poor solubility in our Tween/polysorbate 80-free formulation recipes with or without 5% DMSO. Therefore, future studies related to the structural modification of the FL118 scaffold should be based on the findings revealed from these compounds to further optimize potency and drug-like properties, for example by adding hydrophilic group on positions of 5, 9 or 12. Alternatively, we may generate the pro-drug for potent compounds, such as dipeptide derivatives to increase water solubility. Working in these directions may allow us to generate compounds with even better therapeutic index (TI, i.e. ratio of antitumor activity versus toxicity) than the favorable TI of FL118 [29].

Here, it should be pointed out that the relationship between the affinity of camptothecin analogues to ABCG2 and the strength of electronegative charges of distinct chemical groups on the B ring has not been investigated previously, although the B ring of camptothecin is often modified in order to improve the anticancer potency and pharmacological properties [25,26]. While there was a positive correlation between χ and ABCG2-induced resistance, the large loss of potency for 7-bromomethyl-FL118 compared to 7-chloromethyl-FL118 suggests that functional group electronegativity may be only one characteristic that influences ABCG2 affinity. Since ABCG2-mediated resistance to anticancer drugs, including irinotecan and topotecan, is a recognized problem in the clinic, further understanding of the structure-activity relationship of FL118 analogues for ABCG2 binding may lead to rational design of better anticancer agents for clinical application.

Interestingly, cabazitaxel, a taxane derivative with poor affinity for another drug-efflux pump protein, P-glycoprotein 1 (P-gp, also known as multidrug resistance protein 1 and ABCB1), was recently approved for use in patients with castration-resistant prostate cancer who had previously failed docetaxel-based regimens [30]. It is thought that cabazitaxel’s lack of affinity for P-gp plays an important role in its effectiveness in docetaxel-refractory cancer. In keeping with this rationale, the work presented here suggests that this strategy may be useful in other cancer types and with other classes of cytotoxic agents, including camptothecins. Due to FL118’s superior anticancer activity, favorable tolerability, and insensitivity to ABCG2, we posit that FL118 may become a better option for targeted cancer therapeutics to address the increasingly complex issue of drug failure by circumventing multiple mechanisms of drug resistance, including efflux pump-mediated resistance.

In conclusion, the present study demonstrated that FL118 has additional mechanistic features in terms of its superior anticancer efficacy, which further distinguish it from irinotecan, SN-38 and topotecan. These findings suggest that FL118 is a poor substrate for the drug efflux pump ABCG2, and thus FL118 is able to overcome ABCG2-mediated resistance to SN-38, irinotecan and topotecan *in vitro* and *in vivo*. Additionally, this study also indicated that polar chemical groups on the B ring of FL118 analogues can contribute to ABCG2-mediated resistance, which provides one principle for new FL118 analogue design. Together, the new features of FL118 revealed in this study plus the other FL118 unique features reported in our previous studies warrants FL118 further development toward clinical application.

## Materials and methods

### Drug resource and preparation

Topotecan (Selleckchem Chemicals, Houston, TX), FL118 (in house), and FL118 analogues (RTI International) were prepared as stocks at 1 mM in DMSO (Merck KGaA, Darmstadt, Germany). The synthesis of FL118 and FL118 analogues (7-methyl-FL118, 7-ethyl-FL118, 7-allyl-FL118, 7-bromomethyl-FL118, 7-chloromethyl-FL118 and 7-hydroxymethyl-FL118) were reported previously [21,31,32]. Stock SN-38 (Sigma-Aldrich Corporation, St. Louis, MO) was prepared at 2.5 mM in DMSO. Ko143 (Tocris Bioscience, Bristol, United Kingdom) was prepared as stock solutions at 10 mM in DMSO.

### Cell culture

Human colorectal cancer cell lines HCT8, HCT116, and SW620 and NSCLC cell lines A549 and NCI-H460 (“H460”) were purchased from American Type Culture Collection (ATCC, Manassas, VA). The human NSCLC cell line EKVX (donated by Dr. Daniel Chan) was originally from the National Cancer Institute [33]. Camptothecin resistant sub-lines of HCT116 with mutated *Top1* (HCT116-SN6, HCT116-G7, HCT116-A2, and HCT116-SN50) were established and described previously by Drs. Gongora and Del Rio [10]. Drug resistant cell lines were passaged in 10 nM SN-38, except for five days prior to all experiments, to maintain resistant phenotypes. Human embryonic kidney HEK293 cells that were stably transfected with either an ABCG2 expression vector (HEK293/ABCG2) or an empty vector (HEK293/pcDNA3) were provided by Dr. Wendy Huss, which were originally a gift from Dr. Susan Bates (National Cancer Institute, Rockville, MD). All cell lines were maintained in RPMI-1640 medium supplemented with 10% heat-inactivated fetal bovine serum, 100 U/mL penicillin, and 0.1 μg/mL streptomycin (“complete media”). Cells were cultured in 5 % CO_2_ at 37°C and passaged every 2-4 days.

### Immunoblot analysis

Immunoblot analysis was performed as described previously [34], with minor modifications. Briefly, cells were lysed in radioimmunoprecipitation analysis buffer (50 mM Tris, 150 mM NaCl, 0.1% SDS, 0.5% sodium deoxycholate, 1% Nonidet P-40, 10 μg/mL PMSF, 20 μM leupeptin) and sonicated for 15 s using a sonic dismembrator 100 (Thermo Fisher Scientific, Waltham, MA) to homogenize lysate. Next, lysate was denatured with 5X Laemmli Sample Buffer (5X: 300 mM Tris-HCl pH 6.8, 10% SDS, 50% glycerol, 20% β-mercaptoethanol, 0.05% bromophenol blue) and equal amounts of protein were electrophoretically separated on 10-15% SDS-PAGE gels and electrotransferred onto 0.2 μm nitrocellulose membranes (Bio-Rad Laboratories, Inc., Hercules, CA). Membranes were blocked for 1 h at room temperature in 5% skim milk, then incubated with primary antibody (1:1,000 for ABCG2 and 1:5,000 for actin) in 5% bovine serum albumin in TBS-T overnight at 4°C. Membranes were washed with TBS-T, then incubated with species-specific anti-IgG antibodies conjugated to horseradish peroxidase (1:5,000, second antibody) at room temperature for 1 h in 5% milk. Membranes were again washed with TBS-T. Chemiluminescence with ECL plus (PerkinElmer, Inc., Waltham, MA) was used to detect protein using X-ray film (Midsci, St. Louis, MO).

### Cell proliferation and viability assay

Cells were plated in 6-well plates at densities ranging from 2 x 10^5^ to 4 x 10^5^ cells per well, depending on doubling time. On day 2, cells were treated with varying concentrations of indicated compounds in complete media. Final concentration of the vehicle, DMSO, was 0.1% in all treatments with or without drugs. On day 5, attached cells were harvested with 0.5 mL of 0.25% Trypsin-EDTA. Trypsin was deactivated by addition of 0.5 mL of complete media and cells were analyzed on a Vi-CELL XR Cell Viability Analyzer (Beckman Coulter Inc., Brea, CA). EC_50_ values and coefficient of determination (R^2^) were calculated from seven doses of each analyzed compound, in addition to the vehicle control, using GraphPad Prism 6 (GraphPad Software, Inc., La Jolla, CA). Each experiment was performed at least 3 times.

### Transduction of lentiviral particles containing ABCG2-specific or control shRNA

Lentiviral particles containing ABCG2-specific shRNA, with sequences of AACTCTTGAATGACCCTGT (V3LHS_380805) or ATAATACTTGGTAACATCC (V3LHS_380806), and one control non-silencing shRNA (Dharmacon, Lafayette, CO) were prepared in the Rowell Park Cancer Institute shRNA Core Facility as previously described [18]. HCT116-SN50 cells were plated in 6-well plates at a density of 7 x 10^5^ cells/well. The next day, media was aspirated and 0.5 mL of fresh complete media and 0.5 mL of viral supernatant were added to each well. Cells were incubated with viral supernatant in 5% CO_2_ at 37°C for 16 h. Stably transduced cells were selected and maintained in 1 μg/mL puromycin (Invivogen, San Diego, CA).

### Electrostatic potential maps

Electrostatic potential maps were generated using the PBEQ solver module [35] in CHARMM-GUI [36] in default conditions, using protein data bank (.pdb) files and Tripos Mol2 (.mol2) files created with MarvinSketch 14.7.7.0 (ChemAxon, Ltd., Budapest, Hungary). Maps were visualized with PyMOL 1.3r1 edu (Schrödinger, LLC, New York, NY).

### Pauling electronegativity

Electronegativities were taken from published studies [37-40]. When electronegativities for certain functional groups were not reported in those studies, they were calculated using the methods developed by the authors in the cited works [37-40].

### Establishment of human xenograft models

Xenografts were established in female 12-week old severe combined immunodeficiency (SCID) mice. Cells (4 x 10^6^ per injection) were suspended in 200 μL of a 1:1 solution of ice-cold serum-free RPMI 1640 media and matrigel (Corning Incorporated, Corning, NY) and injected subcutaneously into the left flank. When tumors reached an average volume of 100 mm^3^, animals were randomly assigned to one of two treatment groups. One group received 100 mg/kg irinotecan (Camptosar) (Pfizer, New York, NY), the MTD, IP once per week. The other group received the MTD of FL118, 1.5 mg/kg, IP once per week. The treatment schedule, one treatment per week for 4 weeks, followed by 1 week of rest, repeated until progression, was selected to approximate the administration of camptothecin-class drugs in the clinic [41,42]. Tumor volume and body weight were measured three times per week. Progression was defined as a tumor volume ≥ 1500 mm^3^ or a moribund condition. Tumor volume was calculated as V = 0.5*(length x width^2^), and was measured using digital calipers.

### Statistical analysis

An extra sum-of-squares F test was used to compare dose response curves. Comparison of survival curves for xenograft models was done using the log-rank test. To assess the difference between calculated RR values in camptothecin-resistant HCT116 sublines, which had logarithmic error, the RR value was back-transformed to LogRR so that the error would be symmetric. The LogRR values for FL118 and SN-38 were then compared using Student’s t-test. A p-value of ≤ 0.05 was considered significant for all analyses. Power analysis to determine appropriate group sizes for *in vivo* work was done with the following parameters: α = 0.05, power = 0.8.

### Study approval

All studies using animals were approved by the Institutional Animal Care and Use Committee (IACUC) at Roswell Park Cancer Institute.

## Additional file

Additional file 1: Figure S1. Structure of camptothecin, FL118, and clinically relevant camptothecin analogues (irinotecan, SN-38, topotecan) is shown. The conventional ring nomenclature of camptothecin is denoted. **Figure S2.** Dose-response graphs for FL118 in a panel of cancer cell lines. Cells analyzed after 72-hour treatment. Viability for each dose was determined using a ViCELL XR cell viability analyzer and normalized to that of DMSO control. EC50 and coefficient of determination (R2) calculated using GraphPad. Error bars = SEM, n = 3 independent experiments. **Figure S3.** Dose-response graphs for SN-38 in a panel of cancer cell lines. Cells analyzed after 72-hour treatment. Viability, EC50, and R2 were determined as in Figure S2. Error bars = SEM, n = 3 independent experiments. **Figure S4.** Pharmacological inhibition of ABCG2 modulates potency of topotecan. Dose-response curve of topotecan in the presence and absence of 1 μM Ko143 after 72-hour treatment is shown. Viability determined as in Figure S2. Error bars = SEM, n = 3 independent experiments. **Figure S5.** Electrostatic potential maps of FL118 and its analogues. Maps were generated in the PBEQ solver module of the CHARMM-GUI and visualized using PyMOL. The red and blue gradient in each molecule as shown represents areas with least and most electrostatic potential, respectively. The green arrows next to 7-ethyl-FL118 and 7-chloromethyl-FL118 point toward the added functional group and highlight the difference in localized electrostatic potential discussed in the text. **Table S1.** LogEC50 and LogRR of FL118 and SN-38 in HCT116 colorectal cancer cell lines and Top1 inhibitor-resistant HCT116 sub-lines. **Table S2.** EC50 of FL118 and SN-38 in cells with genetically altered ABCG2 expression.

## Abbreviations

NSCLC: non-small cell lung cancer
Top1: Topoisomerase I
ABC: ATP-binding cassette protein
IP: Intraperitoneal
IV: Intravenous
RR: Relative resistance
TI: Therapeutic index
