# Supplementary material for: FL118, a novel camptothecin derivative, is insensitive to ABCG2 expression and shows improved efficacy in comparison with irinotecan in colon and lung cancer models with ABCG2-induced resistance
Source: Mol Cancer. 2015 Apr 28;14:92. doi: 10.1186/s12943-015-0362-9 (PMC4427926; doi:10.1186/s12943-015-0362-9)
Supplement: Supplementary file 1 [file 12943_2015_362_MOESM1_ESM.doc]

Figure S1: Structures of camptothecin, FL118, and clinically relevant camptothecin analogues

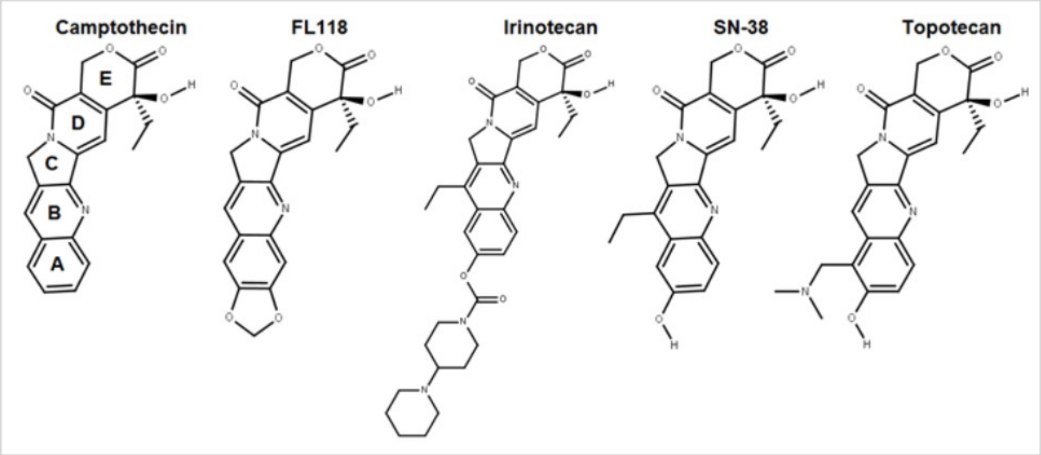

Figure S2: Dose-response graphs for FL118 in a panel of cancer cell lines

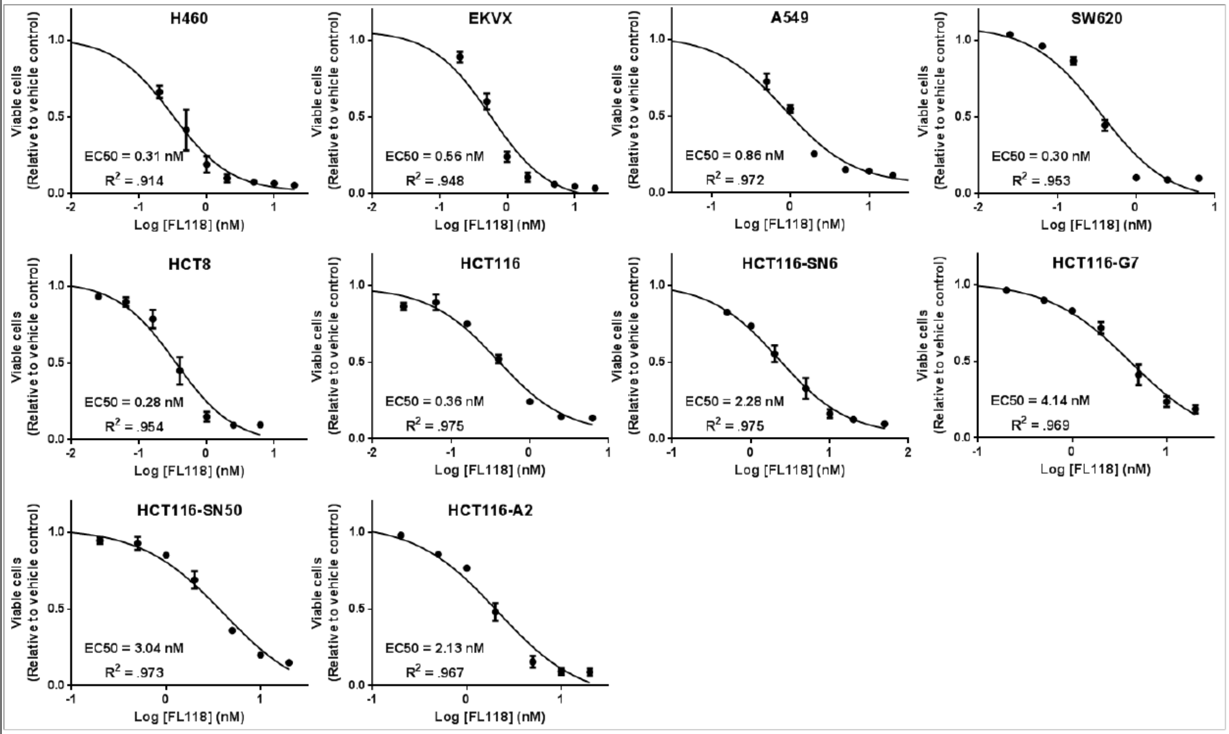

Figure S3: Dose-response graphs for SN-38 in a panel of cancer cell lines

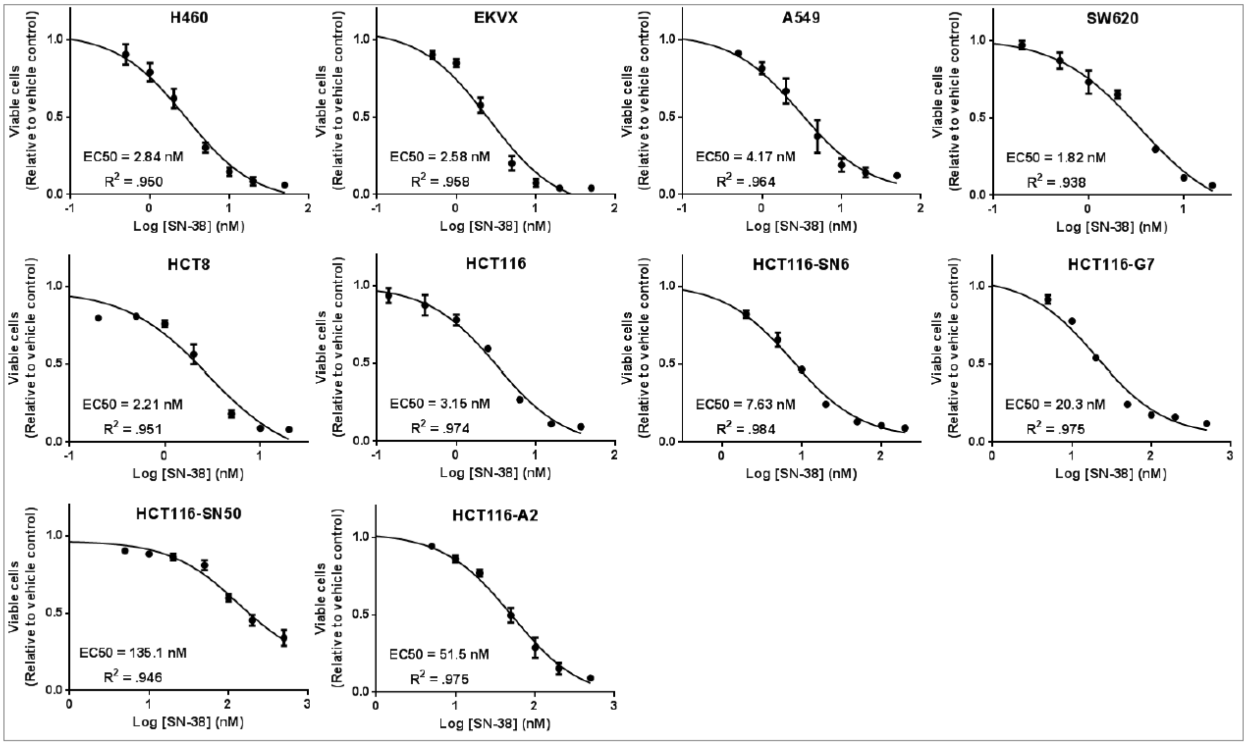

Figure S4: Pharmacologic inhibition of ABCG2 modulates potency of topotecan

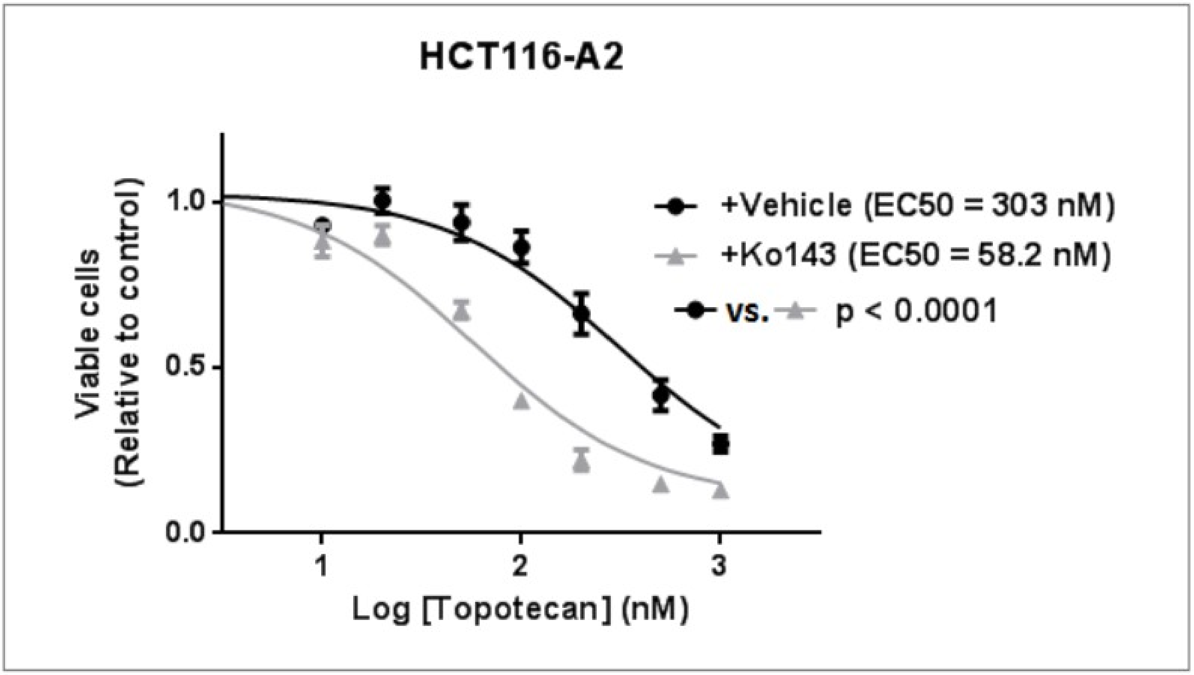

Figure S5: Electrostatic potential maps for FL118 and analogues

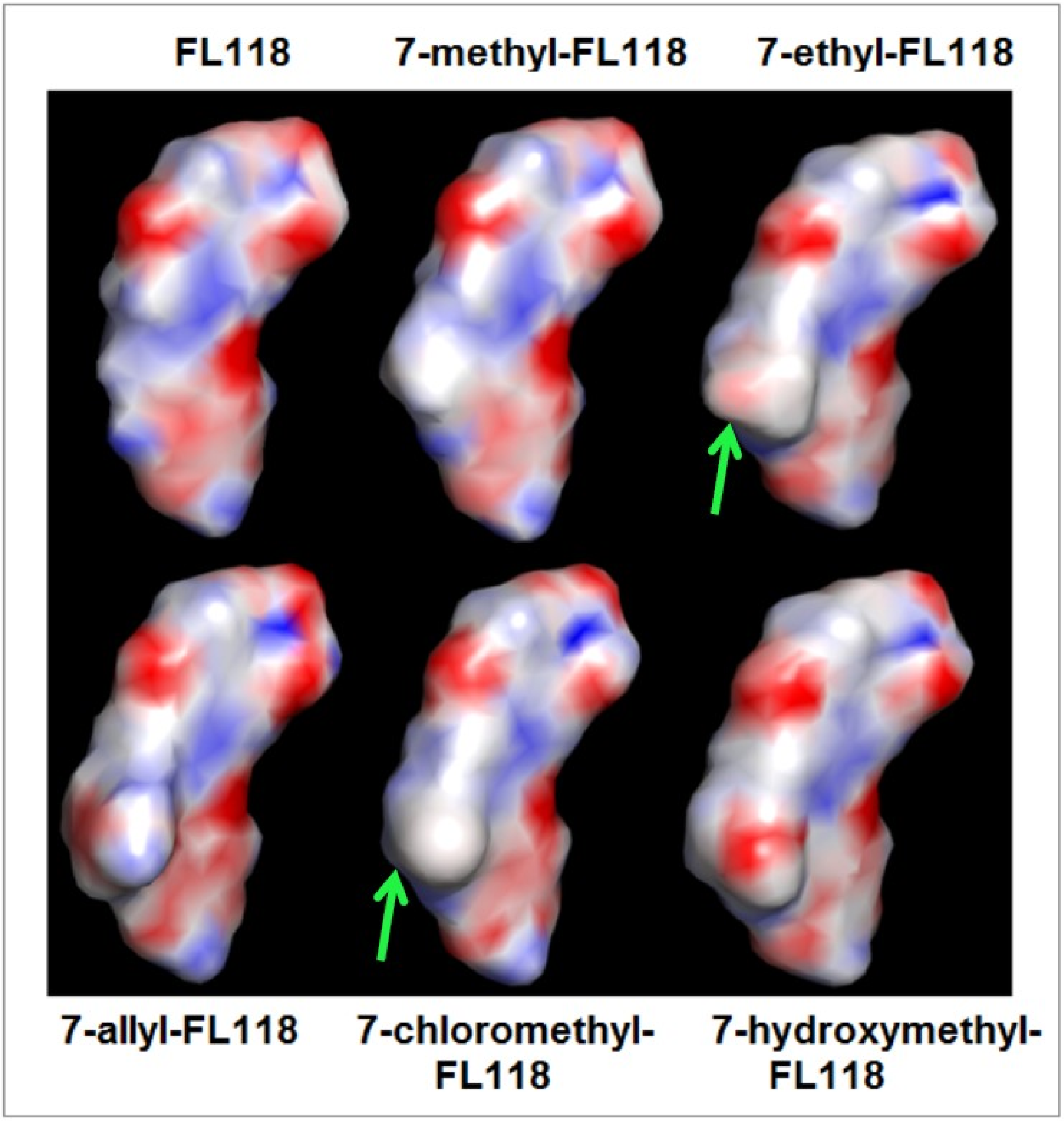

Table S1. LogEC50 and LogRR of FL118 and SN-38 in HCT116 colorectal cancer cell lines and Top1 inhibitor-resistant HCT116 sub-lines

Cell line	FL118 LogEC50 (nM) (± Std. error)	SN-38 LogEC50 (nM) (± Std. error)	FL118 LogRR (± Std. error)	SN-38 LogRR (± Std. error)	*P*
HCT116	-0.439 (± 0.061)	0.498 (± 0.056)			
HCT116-SN6	0.375 (± 0.061)	0.883 (± 0.045)	0.796 (± 0.086)	0.385 (± 0.072)	0.022
HCT116-G7	0.617 (± 0.083)	1.309 (± 0.076)	1.056 (± 0.103)	0.811 (± 0.094)	0.154
HCT116-SN50	0.607 (± 0.078)	2.131 (± 0.108)	1.046 (± 0.099)	1.632 (± 0.130)	0.023
HCT116-A2	0.328 (± 0.076)	1.712 (± 0.067)	0.768 (± 0.098)	1.214 (± 0.087)	0.027

Table S2. EC50 of FL118 and SN-38 in cells with genetically altered ABCG2 expression

Cell line	FL118 EC50 (95% CI) (nM)	*P*	SN-38 EC50 (95% CI) (nM)	*P*
HCT116-A2-ns	2.56 (2.07 - 3.18)		97.00 (74.73 - 125.9)	
HCT116-A2-sh1	2.29 (1.65 - 3.18)	0.57	19.60 (15.98 - 24.03)	< 0.0001
HCT116-A2-sh2	2.75 (1.85 - 4.09)	0.73	22.35 (16.54 - 30.20)	< 0.0001
HEK293/pcDNA3	0.12 (0.06 - 0.26)		0.39 (0.13 - 1.19)	
HEK293/ABCG2	0.32 (0.12 - 0.83)	0.09	62.95 (2.27 - 1744)	0.002

95% CI = 95% confidence interval

P, comparing EC50 of drug in indicated cell line to EC50 of same drug in appropriate control cell line
